# Clinicopathological Predictors of Axillary Pathological Complete Response and Its Prognostic Significance in Clinically Node-Positive (cN+), HER2-Positive Breast Cancer Following Neoadjuvant Therapy

**DOI:** 10.3390/medicina62010200

**Published:** 2026-01-18

**Authors:** Şahin Bedir, Uğur Alp Yeşilova, Merve Tokoçin, Burçin Çakan Demirel, Yakup Bozkaya, Abdilkerim Oyman, Murad Guliyev, Hamza Abbasov, Nebi Serkan Demirci, Ezgi Değerli, Gamze Usul, Ebru Şen, Nilüfer Bulut, Gökmen Umut Erdem

**Affiliations:** 1 Department of Medical Oncology, Istinye University Faculty of Medicine, Gaziosmanpaşa Hospital, Istanbul 34250, Turkey; dr_yakupbozkaya@hotmail.com (Y.B.); droyman84@gmail.com (A.O.); 2 Department of Medical Oncology, University of Health Sciences, Basaksehir Cam and Sakura City Hospital, Istanbul 34480, Turkey; uguralpyesilova@gmail.com (U.A.Y.); ferlut@gmail.com (N.B.); gokmenumut@hotmail.com (G.U.E.); 3 Department of General Surgery, University of Health Sciences, Bagcilar Training and Research Hospital, Istanbul 34100, Turkey; mervetokocin@gmail.com; 4 Department of Medical Oncology, Istanbul Medipol University, Medipol Mega University Hospital, Istanbul 34214, Turkey; burcin.cakandemirel@gmail.com; 5 Department of Medical Oncology, Cerrahpasa Medical Faculty, Istanbul University-Cerrahpasa, Istanbul 34098, Turkey; drguliyev892@gmail.com (M.G.); hamzaabbasov90@gmail.com (H.A.); drserkannebi@yahoo.com (N.S.D.); 6 Department of Medical Oncology, University of Health Sciences, Bakırkoy Dr. Sadi Konuk Training and Research Hospital, Istanbul 34147, Turkey; ezgitastan.19@hotmail.com; 7 Department of Pathology, University of Health Sciences, Basaksehir Cam and Sakura City Hospital, Istanbul 34480, Turkey; gamzeusul89@gmail.com; 8 Department of General Surgery, University of Health Sciences, Basaksehir Cam and Sakura City Hospital, Istanbul 34480, Turkey; ebrusenoran@windowslive.com

**Keywords:** HER2-positive breast cancer, neoadjuvant therapy, axillary pathological complete response, prognostic factors

## Abstract

*Background and Objectives*: This study aimed to identify clinicopathological factors associated with axillary pathological complete response (ApCR) in patients with HER2-positive breast cancer presenting with clinically node-positive disease (cN+) confirmed by biopsy who received neoadjuvant therapy (NAT), and to assess the prognostic significance of ApCR on survival outcomes. *Materials and Methods*: A total of 221 patients with clinically node-positive (cN+) HER2-positive invasive breast cancer, with nodal involvement confirmed by fine-needle aspiration or core needle biopsy, who received neoadjuvant therapy (NAT) and subsequently underwent surgery at three centers between January 2015 and January 2025 were retrospectively reviewed. The association between clinicopathological factors and axillary pathological complete response (ApCR) was analyzed using logistic regression. Survival analyses were performed using the Kaplan–Meier method. *Results*: The median follow-up duration was 34.3 months. Axillary pathological complete response (ApCR) was achieved in 67.9% of patients. The ApCR rate was higher in stage II disease compared with stage III (76.9% vs. 62.9%). Patients with HER2 3+ tumors demonstrated a higher ApCR rate (70.8%) than those with HER2 2+/FISH+ tumors (46.2%). In multivariable logistic regression, HER2 3+ status (OR = 2.745; 95% CI: 1.138–6.619; *p* = 0.025) and lower clinical stage (OR = 2.251; 95% CI: 1.182–4.287; *p* = 0.014) were independently associated with a higher likelihood of achieving ApCR. In survival analyses, the 3-year event-free survival rate was 92% (95% CI: 86–98%) in the ApCR group, compared with 75% (95% CI: 63–87%) in the non-ApCR group. Kaplan–Meier analysis demonstrated that ApCR was a significant prognostic factor for EFS (*p* = 0.001). Median overall survival (OS) was not reached in either group due to the limited number of death events. *Conclusions*: ApCR was frequent in node-positive HER2-positive breast cancer after neoadjuvant therapy. HER2 3+ status and lower clinical stage independently predicted ApCR, which in turn was associated with improved event-free survival. These findings underscore the prognostic relevance of ApCR in this setting.

## 1. Introduction

Breast cancer is the most commonly diagnosed malignancy among women worldwide and remains one of the leading causes of cancer-related mortality [1]. The human epidermal growth factor receptor 2 (HER2) is overexpressed or amplified in approximately 15–20% of newly diagnosed breast cancer cases [2]. HER2-positive breast cancer represents a distinct clinical subtype characterized by aggressive tumor biology and an unfavorable prognosis. In this patient population, particularly among those with axillary lymph node involvement, neoadjuvant systemic therapy (NAT) has become the standard of care [3].

Pathological complete response (pCR), which reflects the efficacy of NAT, is recognized as a strong prognostic indicator of long-term survival, particularly in patients with HER2-positive breast cancer [4]. Beyond improving oncologic outcomes, NAT has also transformed surgical management by increasing the rates of breast-conserving surgery and reducing morbidity associated with axillary procedures [5,6,7]. In patients achieving axillary pathological complete response (ApCR), the feasibility of less invasive approaches, such as sentinel lymph node biopsy (SLNB), has markedly increased [8,9].

Although patients with HER2-positive breast cancer and lymph node involvement are known to derive substantial benefit from neoadjuvant therapy, most existing studies have primarily focused on pCR in the breast, while detailed assessment of axillary response remains relatively limited [10,11]. Therefore, the present study aimed to identify clinicopathological factors associated with ApCR in patients with HER2-positive breast cancer who presented with clinically node-positive (cN+) disease and received NAT, and to evaluate the prognostic impact of ApCR on survival outcomes.

## 2. Materials and Methods

### 2.1. Study Design and Patient Selection

This retrospective cohort study was conducted by analyzing data from patients with HER2-positive invasive breast cancer who were clinically axillary lymph node–positive (cN+) and received neoadjuvant therapy (NAT) between January 2015 and January 2025 at the Medical Oncology departments of Istinye University Gaziosmanpaşa Hospital, Başakşehir Çam and Sakura City Hospital, and Bağcılar Training and Research Hospital. The participating institutions are high-volume tertiary referral centers, each treating approximately 300–500 newly diagnosed breast cancer cases annually. To ensure a homogeneous study population, strict inclusion and exclusion criteria were applied.

### 2.2. Inclusion Criteria

Patients were eligible if they met all of the following criteria:

1. Histopathologically confirmed invasive breast carcinoma;
2. HER2 positivity confirmed by immunohistochemistry (IHC) or fluorescence in situ hybridization (FISH) (IHC 3+ or IHC 2+ with FISH amplification);
3. Clinical or radiological confirmation of axillary lymph node involvement at diagnosis, with node positivity verified by fine-needle aspiration or core needle biopsy;
4. Receipt of trastuzumab-based neoadjuvant systemic therapy;
5. Underwent surgical treatment following completion of neoadjuvant therapy.

### 2.3. Exclusion Criteria

Patients were excluded if they met any of the following conditions:

1. Presence of distant metastases at diagnosis (cM1);
2. Prior axillary surgery before neoadjuvant therapy;
3. Absence of clinically or radiologically confirmed axillary lymph node involvement at diagnosis;
4. Incomplete clinicopathological data;
5. Follow-up duration of less than 6 months;
6. Patients diagnosed with carcinoma of unknown primary (CUP) syndrome.

### 2.4. Clinical and Pathological Assessment

At diagnosis, all patients underwent mammography, breast ultrasonography, magnetic resonance imaging (MRI), and systemic staging with either thoracic and abdominal computed tomography (CT) or positron emission tomography/computed tomography (PET-CT). Clinical staging was performed according to the American Joint Committee on Cancer (AJCC) 8th edition criteria [12]. HER2, estrogen receptor (ER), and progesterone receptor (PR) expression were assessed using IHC. HER2 IHC 2 + cases were further evaluated using FISH test to identify HER2 status. According to the 2018 American Society of Clinical Oncology/College of American Pathologists (ASCO/CAP) guidelines, HER2 positive tumors were defined as HER2 IHC 2+/FISH+ or HER2 IHC 3+ [13]. ER/PR positive was defined as ≥1% of the tumor cells stained positive by IHC [14]. The Ki67 proliferation index was determined by manual counting in “hot spot” areas and reported as a percentage [15]. 20% was considered to be the cut off value of Ki-67 based on the 2013 St. Gallen consensus [16]. Clinicopathological data, including age, menopausal status, tumor size, histological type, tumor grade, ER/PR status, HER2 status, Ki67 level, administered treatment regimens, and surgical procedures, were retrospectively extracted from patient medical records.

### 2.5. Neoadjuvant Therapy and Surgical Approach

All patients, regardless of disease stage or treating center, received neoadjuvant systemic therapy consisting of sequential anthracycline-based chemotherapy followed by taxane in combination with anti-HER2-targeted therapy, including trastuzumab with or without pertuzumab [17,18]. The high rate of dual HER2 blockade observed in this cohort is partly attributable to the inclusion of a substantial number of patients treated in the later years of the study period, during which pertuzumab became more widely available and reimbursed in routine clinical practice.

Following completion of neoadjuvant therapy, all patients underwent surgical treatment, either breast-conserving surgery or mastectomy. The choice of breast surgery was based on initial tumor burden, clinical response to neoadjuvant therapy, tumor-to-breast size ratio, presence of multifocal disease, and patient preference.

Axillary surgical management was guided by post-treatment clinical and radiological findings, surgeon preference, and institutional practice. In a subset of patients who converted to clinically node-negative (ycN0) status after neoadjuvant therapy, sentinel lymph node biopsy (SLNB) was preferentially performed using a dual-tracer technique (radioisotope and/or blue dye) whenever feasible, with retrieval of at least two sentinel lymph nodes required for adequate assessment. Targeted axillary dissection (TAD), including clipping of metastatic axillary lymph nodes at diagnosis, was performed in all patients for whom SLNB was planned. Axillary lymph node dissection (ALND) was performed in patients with persistent clinically suspicious lymph nodes, inadequate response to therapy, confirmed metastatic involvement of lymph nodes, or in those for whom ALND had been planned at initial presentation.

### 2.6. Pathological Response Assessment

Pathological response was evaluated on surgical specimens obtained after completion of NAT. ApCR was defined as the absence of residual invasive tumor in the axillary lymph nodes (ypN0), regardless of the type of axillary surgery performed (SLNB or ALND). Breast pathological complete response (BpCR) was defined as no evidence of invasive carcinoma in the breast tissue (ypT0/is). pCR was characterized by the absence of invasive tumor in both the breast and axillary lymph nodes (ypT0/is, ypN0) [19].

### 2.7. Adjuvant Therapy

Adjuvant therapy after surgery was administered according to institutional protocols, current international guidelines, and individual patient characteristics. Adjuvant radiotherapy was routinely applied to patients undergoing breast-conserving surgery and to selected patients undergoing mastectomy, based on tumor size, lymph node involvement, and pathological response to neoadjuvant therapy. All patients completed one year of anti-HER2-targeted therapy, and adjuvant endocrine therapy was administered to those with hormone receptor–positive disease.

### 2.8. Follow-Up and Survival Analysis

Patients were routinely followed every 3–6 months after surgery. Data on recurrence and survival were retrospectively obtained from medical records and hospital databases. Median follow-up time was estimated using the reverse Kaplan–Meier method. Event-free survival (EFS) was defined as the time from the initiation of neoadjuvant therapy to the occurrence of the first event, including locoregional or distant recurrence, metastasis, second primary malignancy, or death from any cause. Overall survival (OS) was defined as the time from diagnosis to death from any cause or until the date of the last follow-up visit.

### 2.9. Statistical Analysis

All analyses were performed using SPSS software, version 26.0 (IBM Corp., Armonk, NY, USA). The associations between clinicopathological variables and ApCR were assessed using the Chi-square test or Fisher’s exact test, as appropriate. Continuous variables were compared using either Student’s *t*-test or the Mann–Whitney *U* test, depending on the results of the Kolmogorov–Smirnov test for normality. Variables that showed a significant association with ApCR in univariate analysis were entered into a multivariate logistic regression model. Survival analyses were performed using the Kaplan–Meier method, and differences between survival curves were compared with the log-rank test. Multivariate survival analyses were conducted using the Cox proportional hazards regression model. A two-sided *p* value of less than 0.05 was considered statistically significant.

## 3. Results

### 3.1. Patient Characteristics

A total of 221 patients were included in the study. The median follow-up duration was 34.3 months (95% CI: 30.6–38.0) in the overall cohort. The median age at diagnosis was 49 years (range, 24–79), and 70.1% of the patients had a body mass index (BMI) ≥ 25 kg/m^2^. Regarding menopausal status, 56.6% were premenopausal and 43.4% were postmenopausal. The most common histological subtype was invasive ductal carcinoma, accounting for 92.3% of all cases. Hormone receptor positivity was observed in 63.3% of the patients. The majority of tumors were classified as clinical T1–T2 stage (72%), while cN1 disease was the most frequent nodal status (44.3%). Overall, 64.7% of the patients presented with stage III disease. Most of the patients were in the subgroup characterized by HER2 3+ positivity (88.35%) and a Ki-67 index ≥ 20% (92.3%). Total mastectomy was performed in 57.5% of the patients, while axillary lymph node dissection (ALND) was carried out in 70.6%. Regarding systemic therapy, all patients received chemotherapy regimens containing anthracycline, cyclophosphamide, and taxane. Additionally, 71% of patients received targeted therapy with a combination of trastuzumab and pertuzumab. All demographic and clinicopathological characteristics are summarized in Table 1.

### 3.2. Response to Neoadjuvant Therapy

In the entire cohort, the rate of ApCR following neoadjuvant therapy was 67.9%. During the same period, the rate of BpCR was 53.4%, and the overall pCR was 51.1%.

### 3.3. Univariate Analysis of Factors Associated with ApCR

In univariate logistic regression analysis, ApCR was significantly associated with clinical stage, and HER2 status (*p* < 0.05). In stage 2 patients, the ApCR rate was 76.9%, while in stage 3 patients it was 62.9%. Similarly, more advanced clinical stage significantly reduced the probability of ApCR (OR = 1.963; 95% CI: 1.049–3.673; *p* = 0.035). In patients with HER2 3+ tumors, the ApCR rate was 70.8%, whereas it was 46.2% in those with HER2 2+ tumors. In addition, ApCR rates were significantly higher in HER2 3+ tumors compared to HER2 2+ tumors (OR = 2.659; 95% CI: 1.122–6.299; *p* = 0.026). The results of the univariate analysis are summarized in Table 2.

### 3.4. Multivariate Analysis of Factors Associated with ApCR

In multivariate logistic regression analysis, earlier clinical stage, was significantly associated with a higher likelihood of achieving ApCR (OR = 2.251; 95% CI: 1.182–4.287; *p* = 0.014). HER2 status remained an independent prognostic factor, with HER2 3+ tumors exhibiting approximately a 2.7-fold higher probability of achieving ApCR compared to HER2 2+ tumors (OR = 2.745; 95% CI: 1.138–6.619; *p* = 0.025). The results of the multivariate analysis are summarized in Table 3.

### 3.5. Survival Analysis

During the follow-up period, 28 patients experienced recurrence or distant metastasis, and 14 patients died due to breast cancer. At the time of analysis, median EFS and OS had not yet been reached in either group.

### 3.6. Impact of ApCR on Event-Free Survival and Overall Survival

**Figure 1:**
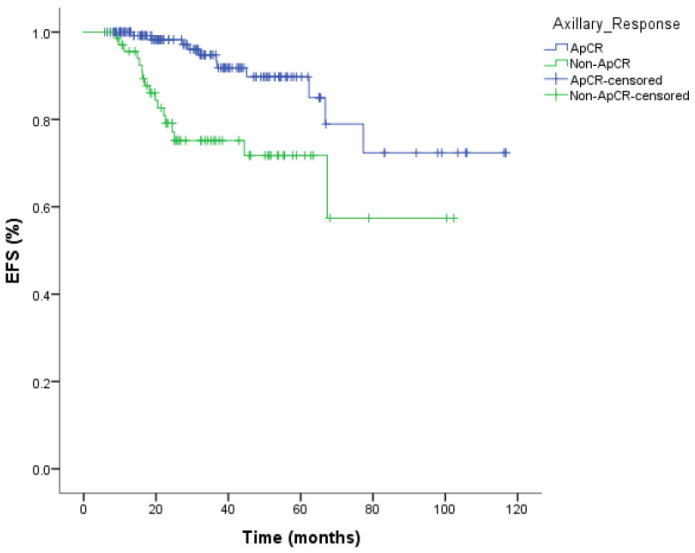
Kaplan–Meier curves for event-free survival (EFS) according to axillary pathological complete response (ApCR) status. Patients achieving ApCR demonstrated significantly improved EFS compared with those without ApCR (log-rank *p* = 0.001).

**Figure 2:**
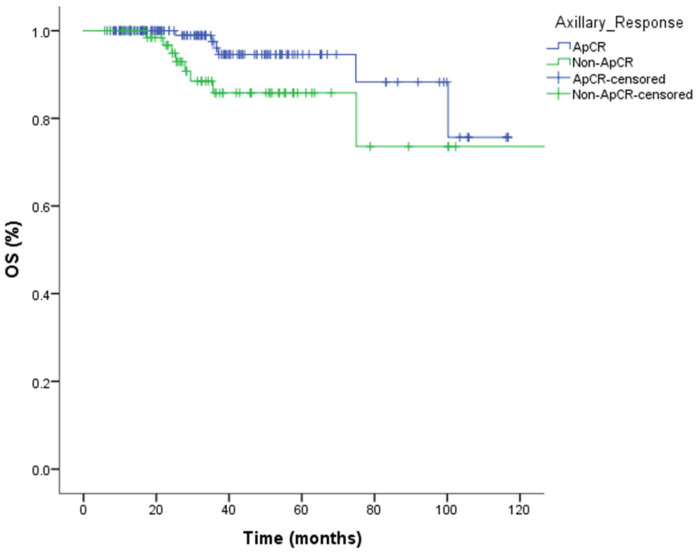
Kaplan–Meier curves for overall survival (OS) according to axillary pathological complete response (ApCR) status. No statistically significant difference in OS was observed between patients with and without ApCR (log-rank *p* = 0.063).

The 3-year event-free survival rate was 92% (95% CI: 86–98%) in the ApCR group, compared with 75% (95% CI: 63–87%) in the non-ApCR group. Kaplan–Meier analysis demonstrated that ApCR was a significant prognostic factor for EFS (*p* = 0.001) (Figure 1). The 3-year overall survival rate was 96% (95% CI: 92–100%) in the ApCR group and 86% (95% CI: 76–96%) in the non-ApCR group. Although patients achieving ApCR demonstrated numerically higher overall survival rates, this difference did not reach statistical significance for OS (*p* = 0.063) (Figure 2), likely due to the limited number of events and insufficient follow-up duration.

## 4. Discussion

In our study, the rate of ApCR was 67.9% among 221 patients with HER2-positive invasive breast cancer who were cN+ at diagnosis and received NAT. Reported ApCR rates in HER2-positive patients vary across studies, ranging from approximately 56.6% to 79.1% [20,21,22]. Our findings are consistent with this range, despite the inclusion of only cN+ patients in our cohort. Differences in ApCR rates can be attributed to variations in baseline nodal status, NAT regimens employed (single versus dual HER2 blockade), hormone receptor status, and heterogeneity in HER2 expression levels across study populations.

Importantly, despite the high ApCR rate in our cohort, SLNB was performed in only 29.4% of the entire cohort and in 36% of patients who achieved ApCR. This observation mirrors previously published data, including the studies by Hamdy et al. and Cha et al., which reported SLNB rates of 28.1% and 26%, respectively [20,23]. These consistently low rates suggest that SLNB adoption in initially node-positive patients remains limited, likely due to concerns about false-negative results, insufficient use of targeted axillary approaches, and variability in institutional surgical practices. Together, these findings highlight ongoing heterogeneity in axillary management after NAT, even in patient groups with a high likelihood of achieving ApCR.

Patients with HER2 IHC 3+ tumors demonstrated a significantly higher likelihood of achieving ApCR compared to those with HER2 IHC 2+/FISH+ tumors. Similarly, the literature reports that ApCR rates in HER2 3+ tumors are approximately 2–5 times higher than in HER2 2+/FISH+ tumors [20,24,25]. These findings support the notion that HER2 3+ tumors, owing to their biologically more homogeneous profile and stronger HER2 expression, exhibit greater sensitivity to anti-HER2 therapies. Additionally, these results suggest that treatment response may be limited in patients with HER2 2+/FISH+ tumors, highlighting the need to explore additional biomarkers or alternative therapeutic strategies for the clinical management of this subgroup. In the DESTINY-Breast11 trial, in which 89% of patients had node-positive disease, the pathologic complete response rate in the HER2 2+/FISH+ subgroup increased to as high as 42.5%, indicating that T-DXd may represent a promising therapeutic option for these patients [22].

Advanced clinical stage was shown to significantly reduce the likelihood of achieving ApCR. Although this finding does not entirely align with previous reports, it shares certain similarities. For instance, Fernández-González et al. found no significant association between clinical stage and ApCR (*p* = 0.894) [21]. In contrast, Chen et al. reported that patients with lower clinical stages had a significantly higher probability of achieving pCR (*p* = 0.0223) [25]. Therefore, differences in patient populations, tumor subtypes, and biological characteristics may contribute to the variability observed among studies regarding the relationship between clinical stage and pathological response.

In our study, achieving ApCR was shown to significantly prolong event-free survival (EFS). This finding is consistent with previous studies emphasizing the prognostic significance of ApCR on survival outcomes. Inari et al. reported that ApCR was associated with improved distant disease-free survival (DDFS), while Ren et al. demonstrated that patients who achieved ApCR had significantly longer disease-free survival (DFS). Similarly, Fernández-González et al. observed that patients who reached ypN0 status after neoadjuvant chemotherapy exhibited superior DDFS compared with those remaining ypN+ [21,26,27].

In contrast, due to the relatively short follow-up period and the limited number of death events, the median OS could not be reached. Nevertheless, previous reports have demonstrated a strong association between ApCR and improved long-term survival. For example, Inari et al. showed that patients who achieved ApCR experienced significantly longer OS, and Fernández-González et al. similarly reported superior OS outcomes in individuals who attained ypN0 compared with those who remained ypN+ [21,26]. Therefore, while our findings reinforce the strong prognostic value of ApCR for EFS, they also suggest that the impact of ApCR on OS in our cohort may become more evident with longer follow-up.

### Strengths and Limitations

This study has several strengths, including a relatively large and homogeneous cohort of HER2-positive, node-positive patients treated with contemporary neoadjuvant regimens, and the inclusion of data from three high-volume centers, which enhances the generalizability of the findings. The detailed pathological evaluation and the combined analysis of both predictive factors and survival outcomes further strengthen the clinical relevance of the results.

However, the retrospective design introduces potential selection bias, and the median follow-up duration may be insufficient to fully evaluate long-term overall survival. Variability in axillary surgical approaches across centers and the limited size of the HER2 2+/FISH+ subgroup also represent methodological constraints that may influence response rates and subgroup analyses. Furthermore, the limited number of locoregional recurrence events precluded a robust analysis of locoregional outcomes according to pathological complete remission status.

## 5. Conclusions

In this cohort of initially node-positive HER2-positive breast cancer patients, a high rate of axillary pathological complete response was observed, with HER2 IHC 3+ tumors and lower clinical stage significantly associated with ApCR. SLNB was performed in less than one-third of patients, highlighting ongoing variability in axillary management. Achievement of ApCR was a strong predictor of event-free survival, suggesting its value as a prognostic marker. Longer follow-up is needed to fully assess its impact on overall survival and guide individualized axillary surgical strategies.

## Figures and Tables

**Table 1:** Demographic and clinical characteristics of patients.

Characteristics	All n = 221 (%)	ApCR n = 150 (%)	Non-ApCR n = 71 (%)
Age			
≤50	130 (58.8)	89 (59.3)	41 (57.7)
>50	91 (41.2)	61 (40.7)	30 (42.3)
Body Mass Index (BMI)			
<25	66 (29.9)	47 (31.3)	19 (26.8)
≥25	155 (70.1)	103 (68.7)	52 (73.2)
Menopausal Status			
Premenopausal	125 (56.6)	87 (58)	38 (53.5)
Postmenopausal	96 (43.4)	63 (42)	33 (46.5)
Pathological types			
Ductal	204 (92.3)	136 (90.7)	68 (95.8)
Other	17 (7.7)	14 (9.3)	3 (4.2)
Tumor grade			
Grade ½	61(27.6)	43 (28.7)	18 (25.3)
Grade 3	160 (72.4)	107 (71.3)	53 (74.7)
cT			
cT1–T2	159 (72)	114 (76)	45 (63.4)
cT3–T4	62 (28)	36 (24)	26 (36.6)
cN			
cN1	98 (44.3)	70 (46.7)	28 (39.4)
cN2–N3	123 (55.7)	80 (54.3)	43 (60.6)
Clinical TNM Stage			
II	78 (35.3)	60 (40)	18 (25.4)
III	143 (64.7)	90 (60)	53 (74.6)
ER			
Negative	83 (37.6)	61 (40.7)	22 (31)
Positive	138 (62.4)	89 (59.3)	49 (69)
PR			
Negative	104 (47.1)	75 (50)	29 (40.8)
Positive	117 (52.9)	75 (50)	42 (59.2)
HR status			
Negative	81 (36.7)	59 (39.3)	22 (31)
Positive	140 (63.3)	91 (60.7)	49 (69)
HER-2			
2+/FISH+	26 (11.65)	12 (8)	14 (19.8)
3+	195 (88.35)	138 (92)	57 (80.2)
Ki-67			
<20	17 (7.7)	11 (7.3)	6 (8.5)
≥20	214 (92.3)	139 (92.7)	65 (91.5)
Breast surgery			
Mastectomy	127 (57.5)	82 (54.7)	45 (63.4)
BCS	94 (42.5)	68 (45.3)	26 (36.6)
Axillary surgery			
SLNB	65 (29.4)	54 (36)	11 (15.5)
ALND	156 (70.6)	96 (64)	60 (84.5)
Targeted therapy			
Trastuzumab	64 (29)	45 (30)	19 (26.8)
Trastuzumab + pertuzumab	157 (71)	105 (70)	52 (73.2)

Data are presented as n (%). ApCR indicates axillary pathological complete response. Clinical staging (cT, cN) followed the AJCC 8th edition. HR positivity was defined as ER and/or PR expression; HER2 status was assessed by IHC with 2+ cases confirmed by FISH. A Ki-67 cutoff of 20% was applied; BCS, SLNB, and ALND denote surgical procedures.

**Table 2:** Univariate logistic regression for factors related to ApCR.

Characteristics	Odds Ratio (95% CI)	*p* Value
Age	1.068 (0.602–1.893)	0.823
≤50		
>50		
Body Mass Index (BMI)	1.210 (0.633–2.313)	0.565
<25		
≥25		
Menopausal Status	1.199 (0.680–2.116)	0.531
Premenopausal		
Postmenopausal		
Pathological types	0.429 (0.119–1.542)	0.195
Ductal		
Other		
Tumor grade	1.247 (0.648–2.400)	0.508
Grade ½		
Grade 3		
cT	1.830 (0.993–3.371)	0.053
cT1–T2		
cT3–T4		
cN	1.344 (0.757–2.386)	0.313
cN1		
cN2–N3		
Clinical TNM Stage	1.963 (1.049–3.673)	0.035
II		
III		
ER	1.527 (0.838–2.780)	0.167
Negative		
Positive		
PR	1.448 (0.818–2.564)	0.204
Negative		
Positive		
HR status	0.692 (0.380–1.262)	0.230
Negative		
Positive		
HER-2	2.659 (1.122–6.299)	0.026
2+/FISH+		
3+		
Ki-67	0.870 (0.308–2.455)	0.792
<20		
≥20		
Targeted therapy	0.853 (0.454–1.602)	0.620
Trastuzumab		
Trastuzumab + pertuzumab		

Univariate logistic regression was used to evaluate factors associated with ApCR, with results reported as odds ratios (ORs) and 95% confidence intervals (CIs). Clinical staging (cT, cN) followed the AJCC 8th edition; HR positivity was defined as ER and/or PR expression, and HER2 status was assessed by IHC with 2+ cases confirmed by FISH. A Ki-67 cutoff of 20% was applied, and *p* values < 0.05 were considered statistically significant.

**Table 3:** Multivariate logistic regression for factors related to ApCR.

Characteristics	Odds Ratio (95% CI)	*p* Value
Clinical TNM Stage	2.251 (1.182–4.287)	0.014
III		
II		
HER-2	2.745 (1.138–6.619)	0.025
2+/FISH+		
3+		

Multivariate logistic regression identified independent factors associated with ApCR, reported as odds ratios (ORs) with 95% confidence intervals (CIs). Clinical TNM staging followed the AJCC 8th edition, and *p* < 0.05 was considered statistically significant.

## Data Availability

The original contributions presented in this study are included in the article. Further inquiries can be directed to the corresponding author.
